# Nonlinear anisotropic constitutive description of the human basilic vein and comparison with the vein of the lower limb

**DOI:** 10.1007/s10237-025-02014-w

**Published:** 2025-10-29

**Authors:** Nikola Petrová, Zbyněk Sobotka, Lukáš Horný, Karel Filip, Jiří Urban

**Affiliations:** 1 Faculty of Mechanical Engineering, Czech Technical University in Prague, Technická 4, 160 00 Prague, Czech Republic; 2 Krajská zdravotní, a.s. - Masaryk Hospital Ustí nad Labem, Sociální péče 3316/12A, 401 13 Ústí nad Labem, Czech Republic; 3 Third Faculty of Medicine, Charles University, Ruská 87, 100 00 Prague, Czech Republic

**Keywords:** Basilic vein, Constitutive model, Great saphenous vein, Hyperelasticity, Vascular access

## Abstract

The number of patients undergoing hemodialysis has been steadily increasing in recent decades. Arteriovenous fistula (AVF) is the gold standard for ensuring vascular access in these patients. Despite the prominent role of AVFs in hemodialysis treatment, their maturation and long-term functionality continue to pose challenges as less than a third of fistulas remain patent without further interventions in a 3-year follow-up. Computational biomechanics has become an essential tool for clarifying mechanical conditions accompanying the pathogenesis of various vascular complications, including suboptimal maturation and AVF stenosis. Constitutive description plays a crucial role in the design of computational models and without it simulations remain only at the rigid tube level. However, literature on the mechanical properties and constitutive modeling of upper extremity veins is lacking. This study aims to fill this gap by characterizing the mechanical properties of the human basilic vein (BV) and comparing it to the great saphenous vein (GSV). Uniaxial tensile tests in two perpendicular directions were used to obtain the mechanical response of the tissue. The results suggest that BVs do not significantly differ from GSVs in their elastic properties expressed by means of the tangent modulus. Overall anisotropy, understood as the difference in elastic moduli obtained in different directions, seems to be reduced in BVs. The 4-fiber family exponential model of the strain energy density function was adopted to fit the experimental data. The model fitted the data well, as suggested by the coefficients of determination *R*^2^, which ranged from 0.97 to 0.99 for the majority of the average curves. The resulting parameter values can be used within the modeling of the mechanical behavior of veins in computational simulations of vascular access performance.

## Introduction

The number of patients undergoing hemodialysis has been steadily increasing in recent decades (Lawson et al. 2020). Boerstra et al. (2024) report over 550,000 patients undergoing kidney replacement therapy across Europe, of which 56% are treated with hemodialysis. The arteriovenous fistula (AVF) procedure, first introduced in 1966 (Brescia et al. 1966), represents a gold standard for facilitating vascular access in hemodialysis in patients with end-stage renal disease (Santoro et al. 2014; Boerstra et al. 2024). AVF is most commonly created on the wrist, where the cephalic vein is surgically connected to the radial artery, or on the forearm, where the median cubital branch of the cephalic vein is connected to the brachial artery (Lawson et al. 2020). With transposition of the basilic vein, an AVF can also be established on the upper arm (Lawson et al. 2020).

The percentage of fistula users among hemodialysis receiving patients is approximately between 60 and 70%, depending on the healthcare system and patients’ comorbidities (United States Renal Data System 2024). Before the AVF matures and when it fails, other types of vascular access have to be used, mainly the central vein catheter (CVC), the introduction of which is relatively fast and straightforward (Noordzij et al. 2014; Robinson et al. 2016; Stel et al. 2024). CVC is then, unlike AVF, ready for immediate use. However, long-term use of these hemodialysis catheters is not recommended due to their high rate of infectious complications and central vein stenosis formation, which then leads to higher morbidity and mortality rates (Schmidli et al. 2018; Balaz and Björck 2015). Despite the prominent role of AVFs in hemodialysis treatment, their maturation and long-term functionality continue to pose challenges as less than a third of fistulas remain patent without further interventions in a 3-year follow-up (Huber et al. 2021; Heindel et al. 2022; Pfister et al. 2023).

The performance of AVFs is influenced by a complex mechanobiology that takes place within the arterialization of a vein. It involves an interplay of biomechanical factors, such as wall shear stress (WSS), blood flow dynamics, and vascular wall compliance (Bozzetto et al. 2024). The creation of vascular access by means of AVF can fail before the actual use due to insufficient maturation during which the vein adapts to arterial conditions, as well as later within long-term use. WSS, in particular, has been closely linked to the development of intimal hyperplasia, a condition that is often associated with fistula failure (Cunnane et al. 2017). Beside thrombosis, other causes of failure can be attributed to inflammation or the development of an AVF aneurysm, for instance (Balaz and Björck 2015; Lawson et al. 2020; Baláž et al. 2020).

Computational biomechanics has become an essential tool for clarifying mechanical conditions accompanying the pathogenesis of various vascular complications, including suboptimal maturation and AVF stenosis (Boghosian et al. 2014; Bozzetto et al. 2016, 2018, 2024; de Villiers et al. 2018). These simulations allow the analysis of hemodynamics in detail, providing insights into how the oscillatory flow pattern and time-varying gradient of WSS contribute to pathological changes in the blood vessel wall (Ventre et al. 2021; Stella et al. 2019; Zhu and Sakai 2021).

Constitutive models, which mathematically express the mechanical properties of tissues, play a crucial role in the design of computational models. Without reliable constitutive models for the venous wall, simulations remain only at the rigid tube level and thus cannot provide quantitative information about wall stress (Decorato et al. 2014; McGah et al. 2014; de Villiers et al. 2018). However, vascular smooth muscle cells (VSMCs) are involved in the adaptation of the vessel wall to changes in the mechanical environment. This happens not only through their contractile function, which is linked to mechanotransduction from the endothelium that responds to changes in shear stress, but also through their proliferation, migration and by synthetic function. In these adaptations, VSMCs participate in the production and/or digestion of the extracellular matrix. Thus, they thereby contribute to the remodeling of the blood vessel wall and to the preservation of homeostasis during the time-varying load-carrying process (Chien 2007; Qiu et al. 2014; Yu et al. 2024).

Thus, experimentally validated constitutive models for the venous wall are essential to further advancing our understanding of the biomechanics of vascular access for dialysis. Somewhat surprisingly, however, the scientific literature on a mathematical description of the mechanical properties of upper extremity veins is relatively sparse. The vast majority of the experimental results we have been able to trace so far has been obtained with veins that serve as grafts for aorto-coronary bypass surgery. The main resource for knowledge of vein biomechanics is the great saphenous vein (GSV) (Donovan et al. 1990; Karimi et al. 2015; Veselý et al. 2015; Prim et al. 2016, 2021; Li 2018a, 2018b). The scientific attention drawn specifically to GSV has been driven by its usage as a small-diameter bypass conduit, and it remains the golden standard for coronary and peripheral bypass surgery (Mallis et al. 2020; Lampridis and George 2021). However, GSV is of a very different anatomical location than the common AVF for vascular access in hemodialysis. There are also various papers available that deal with the biomechanics of the jugular vein (Sassani et al. 2013; Sokolis 2013), vena cava inferior and superior (Weizsäcker 1988; Desch and Weizsäcker 2007; Alastrué et al. 2008; Hernández and Peña, 2016), and the bridging veins, for example (García-Vilana et al. 2021; García-Vilana and Sánchez-Molina 2022). However, literature on the mechanical properties and constitutive modeling of upper extremity veins is lacking.

This study aims to fill this gap by characterizing the mechanical properties of the human basilic vein (BV) and comparing it to the GSV. Uniaxial tensile experiments in two perpendicular directions are used to assess the anisotropic mechanical response of the tissue. The 4-fiber family hyperelastic model introduced in Baek et al. (2007) is adopted to fit the experimental data. The resulting parameter values can be used within the modeling of the mechanical behavior of veins in computational simulations of vascular access performance. To the best of our knowledge, this study is the first to yield a constitutive description of the human basilic vein compatible with the theory of nonlinear elasticity.

## Materials and methods

### Sample acquisition

**Fig. 1:**
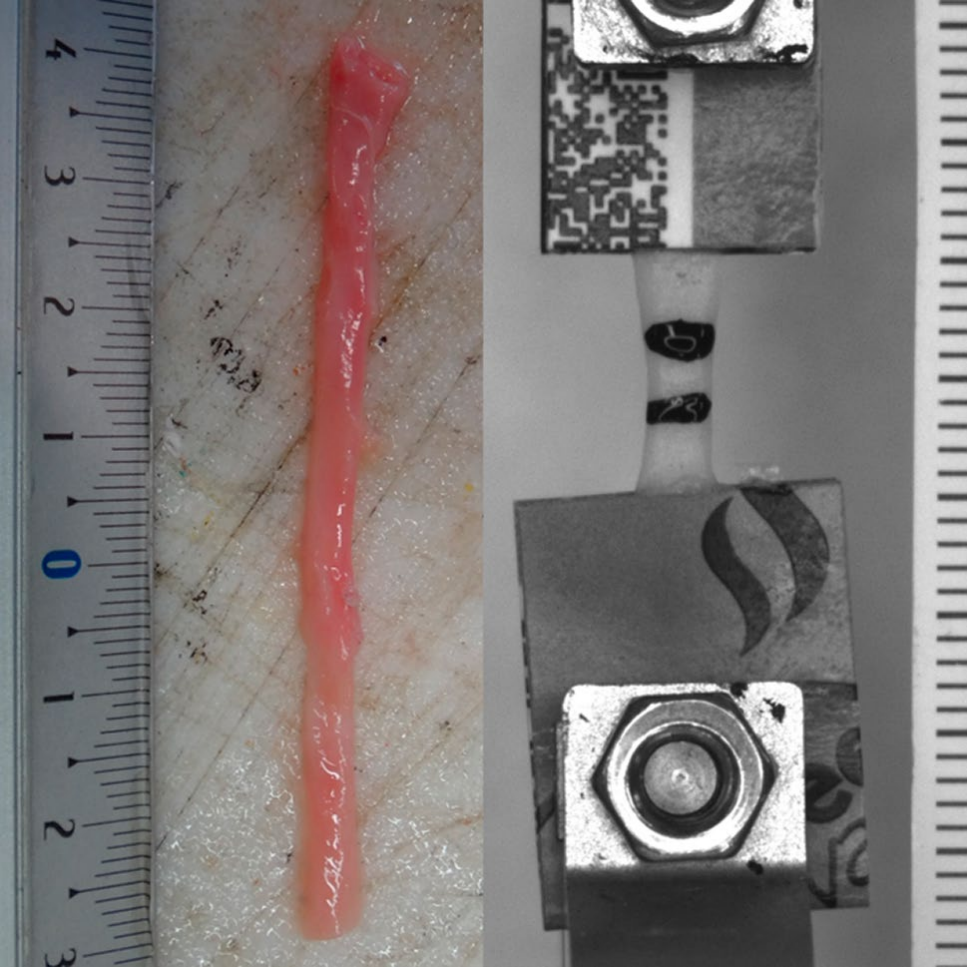
Tested sample. Left—a section of basilic vein obtained from a donor. Right—a strip cut from the vessel, clamped in the testing machine

Samples of the venous tissue were obtained from a total of 10 human donors, with five donors in each group. BVs were collected post-mortem during regular autopsies, while GSV samples were obtained as unused sections of autologous grafts from patients undergoing coronary artery bypass graft surgery. All tissue samples were collected at the Masaryk Hospital in Ústí nad Labem, Czech Republic, and the Ethics Committee of the Masaryk Hospital in Ústí nad Labem approved the use of human tissue in our study. The vein sections were stored in saline at a controlled temperature of 6 °C and then transported to the Czech Technical University in Prague for mechanical experiments. The time between mechanical testing and sample acquisition, or the donor’s death in the case of basilic veins, did not exceed 48 h. The average donor age for each group was 74.2 ± 12 and 69 ± 23 years for basilic veins and GSVs, respectively. The samples were labeled based on the donor’s sex and age, applying the following key: TT_SAA, where TT is the type of vein (BV = basilic vein, SV = saphenous vein), S is the sex of the donor (F = female, M = male), and AA is the age of the donor. A sample, as obtained during surgery, is depicted in Fig. 1. Only veins showing no apparent signs of pathology were included in the study.

### Histological analysis

A small tissue sample was excised from each vein prior to its transport for mechanical testing in order to perform histological analysis. The samples were fixed in 10% formalin and embedded in paraffin blocks. Sections of 3–5 µm thickness were prepared and stained using three different dyes. Hematoxylin–eosin (HE) staining was used as the most common histological dye, which stains VSMCs red, and nuclei dark purple, while collagen appears light pink. Martius, Scarlet and Blue (MSB) staining was used to get a better overview of the vessel wall structure, dying collagen fibers in blue, cell cytoplasm in light green, and nuclei in brown. Elastic fibers are stained dark red with orcein, while mature fibrin appears red, and fresh fibrin yellow to orange. Additionally, Verhoeff van Gieson (VG) staining was used to assess the presence of acidic polysaccharides and some types of mucopolysaccharides in the samples, coloring nuclei and elastic fibers black, muscle fibers yellow, and collagen red. Alcian blue is used to stain acidic polysaccharides and some types of mucopolysaccharides.

### Testing protocol and statistical analysis of stiffness

Three to four thin strips were cut from each sample in both circumferential and longitudinal directions. The thickness of the strips was determined using a bench micrometer based on the average of three measurements taken along the specimen’s length. The samples were kept in saline solution until clamped in the testing machine (see Fig. 1). Uniaxial tensile tests were then carried out with the strips. The tensile tests were performed on a multipurpose testing machine (Zwick/Roell, Germany) equipped with a built-in video extensometer. HBM U9C (accuracy class 0.2) force transducers (Hottinger Brüel and Kjær, Germany) with a measurement range of ±25 N were employed to measure the applied forces. A 5 Mpx, 20 Hz u-Eye camera (IDS, Germany) captured images of the samples during the experiments. Longitudinal deformation of the strips was assessed in real-time using the measurement software TestExpert II 3.5 (Zwick/Roell, Germany), based on images recorded by the video extensometer, utilizing an edge detection algorithm. The testing protocol included five preconditioning cycles in a loading range of 0–0.5 N with a constant crosshead velocity of ±0.1 mm/s. The loading phase of the sixth cycle was used for subsequent analysis, including constitutive modeling. The experiments were carried out in air at room temperature, and no testing procedure lasted longer than two minutes.

A statistical analysis comparing stiffness between the groups was carried out. An initial and a tangent Young’s modulus was determined for each specimen. The initial Young’s modulus *E*_ini_ was computed based on measured stretch and stress in the interval 0–0.5 kPa. The tangent Young’s modulus *E*_100_ was determined for the stress value 100 kPa.

Linear mixed-effect models were employed to account for repeated measurements from multiple specimens obtained from the same donor. These models included vessel type (BV and GSV) as a fixed effect and donor ID as a random effect. This allowed to model intersubject (between-donor) and intrasubject (within-donor) variability separately and estimate group-level elastic moduli while accounting for donor-specific effects. By using these models, it was also possible to distinguish between the donor-level and group-level variance, since the vessel samples of different type did not come from the same donors. The models were fit using restricted maximum likelihood (REML) with the statsmodels library in Python. For both stress levels and directions, the mean modulus and standard deviation were reported using the fitted values from the mixed-effects model, which reflects group-level estimates rather than raw averages.

### Constitutive model

The passive mechanical behavior of the venous tissue was considered to be hyperelastic and incompressible for the purpose of the constitutive modeling, which is an assumption commonly made for blood vessels, given that preconditioning has been performed (Fung 1993; Holzapfel and Ogden 2010; Weisbecker et al. 2015; Holzapfel et al. 2015; Kamenskiy et al. 2017; Li 2018a). The mechanical response of an incompressible hyperelastic material can be described by means of the strain energy density function *W* that plays a role in elastic potential. In such a case, the relationship between stress and strain can be expressed through Eq. (1).

1 $${\mathbf{P}} = \frac{{\partial W({\mathbf{F}})}}{{\partial {\mathbf{F}}}} - p{\mathbf{F}}^{{ - {\text{T}}}}$$

Here, **P** is the first Piola–Kirchhoff stress tensor, and **F** is the deformation gradient. The term *p* is an undetermined multiplier representing the hydrostatic stress component, which does not contribute to *W*, due to the incompressibility of the material. The value of *p* must be determined from the force boundary conditions. For a comprehensive overview of the theory of hyperelasticity, the reader can refer to Ogden (1997), Bonet and Wood (1997), Holzapfel (2000), Truesdell et al. (2004), and Itskov (2019). Details of application within the context of the blood vessels’ biomechanics can be found, for instance, in Humphrey (2002), Taber (2004, 2020), or Gasser (2021).

The model used in this study is a form of an anisotropic exponential model based on the Holzapfel–Gasser–Ogden model (Holzapfel et al. 2000). It was first introduced by Baek et al. (2007) and has commonly been used to model blood vessel tissue (Zeinali-Davarani et al. 2009; Kamenskiy et al. 2017; Jadidi et al. 2020). The particular expression for the strain energy density function is defined in (2).

2 $$W = \frac{\mu }{2}(I_{1} - 3) + \sum\limits_{j = 1..4} {\frac{{k_{1}^{j} }}{{4k_{2}^{j} }}} (e^{{k_{2}^{j} (I_{4}^{j} - 1)^{2} }} - 1)$$

Here, *W* is formulated as a function of the deformation invariants. The first term in (2) represents the isotropic neo-Hookean model, the simplest model of strain energy density fully compatible with finite strain theory. *I*_1_ refers to the first principal invariant of the right Cauchy–Green strain tensor **C**, **C** = **F**^T^**F**, and *μ* is the stress-like constitutive parameter. The anisotropy of the model (2) is incorporated by assuming four preferred directions defined by structural unit vectors ***M***^*j*^ (*j* = 1, …, 4). The preferred directions are considered to lie in the cylindrical surfaces of the vessel, and their components can be in cylindrical polar coordinates (*R*,Θ,*Z*) expressed as ***M***^*j*^ = (0, cos(*β*^*j*^), sin(*β*^*j*^))^T^ with *β*^*j*^ being the angle between the *j*-th preferred direction and the circumferential axis. The invariants *I*_4_^*j*^ introduced in (2) then can be obtained as *I*_4_^*j*^ = ***M***^*j*^·**C*M***^*j*^. Each of the four anisotropic terms in (2) contains a stress-like parameter *k*_1_^*j*^, a dimensionless parameter *k*_2_^*j*^*,* and the angle *β*^*j*^*.* In this study, it is assumed that the first and second preferred directions are aligned in the circumferential (*β*^1^ = 0) and the longitudinal (*β*^2^ = 90°) directions, respectively. The third and fourth preferred directions are set to be symmetric with respect to the axis of the tube. Thus, only one angle *β* = *β*^3^ = −*β*^4^ acts as a constitutive parameter in Eq. (2). Furthermore, constraints *k*_1_^4^ = *k*_1_^3^, and *k*_2_^4^ = *k*_2_^3^ were applied, reducing the number of independent constitutive parameters to eight (*µ*, *k*_1_^1^, *k*_2_^1^, *k*_1_^2^,* k*_2_^2^, *k*_1_^3^, *k*_2_^3^, *β*). It is worth mentioning that the model is inspired by the structural characteristics of blood vessels (Holzapfel et al. 2000). However, in this study, the model is used as a purely phenomenological one. The aforementioned constitutive assumptions should not be confused with the real histological arrangement of the venous wall.

### Regression analysis

Data from the three to four experiments with strips in the circumferential and longitudinal directions were averaged to form a pair of representative loading curves for each of the 10 vein samples. These curves were constructed in the range of nominal stresses 0–200 kPa to ensure the averaged data fell within the preconditioned region of loading. These representative curves were then fitted by the model. The constitutive parameter values were estimated using the weighted least squares method by minimizing the objective function *Q*, formulated in Eq. (3). Here, *P*_*θΘ*_^EXP^, *P*_*θΘ*_^MOD^, *P*_*zZ*_^EXP^, and *P*_*zZ*_^MOD^ refer to the experimental and model-predicted stresses for given stretches in the circumferential and longitudinal directions, respectively. The term *m*, the number of points from which each curve was constructed, was selected as 20. Model stresses were computed from the measured stretches according to Eq. (1). Due to the video extensometer occasionally producing artifacts in the measurements of transverse deformation, the measured transverse deformation was not directly substituted into Eq. (1). Instead, since a uniaxial stress state is assumed, it was treated as an unknown variable, and its values were determined by applying the condition of zero transverse stress at each loading step. This was achieved by applying constraints (4) and (5) on the transverse stresses of the circumferential and longitudinal strips, respectively. Thus, the optimal constitutive parameter value estimates are obtained by solving a constrained optimization problem that minimizes (3), subject to constraints (4) and (5).

3 $$Q = \sum\limits_{i = 1}^{m} {\left[ {\left( {P_{\theta \Theta }^{{{\text{EXP}}}} - P_{\theta \Theta }^{{{\text{MOD}}}} } \right)_{i}^{2} + \left( {P_{zZ}^{{{\text{EXP}}}} - P_{zZ}^{{{\text{MOD}}}} } \right)_{i}^{2} } \right]}$$

4 $$P_{zZ,i}^{{{\text{MOD}}}} = 0 \, i = 1 \ldots m$$

5 $$P_{\theta \Theta ,i}^{{{\text{MOD}}}} = 0 \, i = 1 \ldots m$$

The optimization routine was performed using the GlobalSolve procedure available in Maple 2023 (Maplesoft, Canada), which combines genetic algorithms and local solvers to find the global minimum of a function. The coefficient of determination, *R*^2^, was used to determine the goodness of fit to the experimental data.

## Results

A total of 68 strips cut from 10 tissue samples of human BVs and GSVs (five samples in each group) were obtained for this study. At least three specimens were created in both circumferential and longitudinal directions from every sample. Five experimental measurements were unsuccessful due to handling errors or issues during the experiment, such as false detection of the marker by the video extensometer. Hence, 63 experiments were successfully conducted, with at least two measurements for every vein sample in each direction.

### Stress–strain curves

**Fig. 2:**
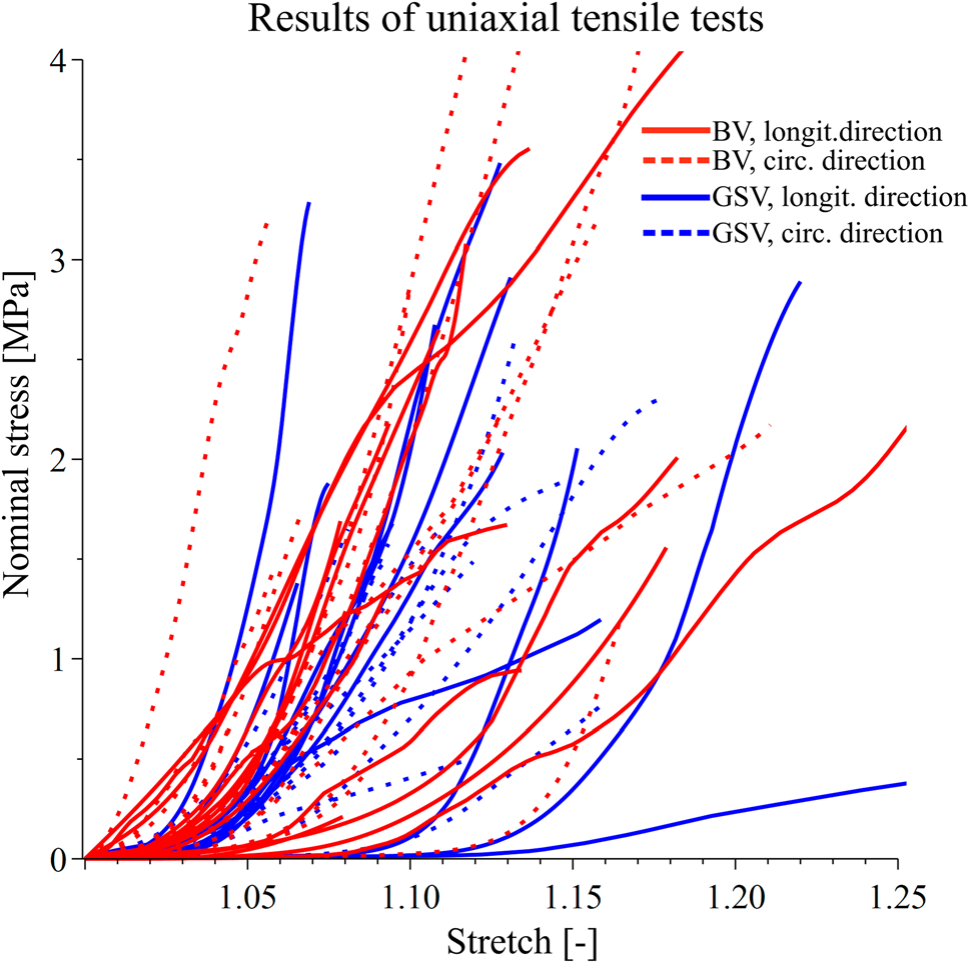
Results of uniaxial tensile test performed with human basilic (red) and great saphenous (blue) veins carried out with longitudinal (solid line) and circumferential (dotted) strips

The results of the uniaxial tensile tests are shown in Fig. 2. It is clear that both tested groups display a qualitatively similar nonlinear, anisotropic behavior typical for blood vessels, although minor differences in overall compliance can be observed. The mean values of the Young’s modulus obtained using linear mixed-effect models for every sample group and direction are shown in Table 1. Although the BV exhibits higher initial stiffness on average, the difference was not significant at the 95% confidence level between any of the specimen groups for either *E*_ini_ or *E*_100_. The most significant difference was found in *E*_ini_ between the longitudinal and circumferential specimens of GSV (*p* = 0.065). In all other comparisons for both *E*_ini_ and *E*_100_ in either strip direction, the *p* value was always higher than 0.1. The results thus suggest that BVs do not significantly differ from GSVs in their elastic properties, expressed by means of the tangent modulus in corresponding directions. On the other hand, overall anisotropy, understood as the difference in elastic moduli obtained in different directions, seems to be reduced in BVs. This is suggested by the standard deviations (SD) in Table 1, where the responses of BVs are accompanied by higher variances.

**Table 1:** Young’s modulus of the sample groups (mean ± SD)

Sample group	BV longit. (MPa)	BV circ. (MPa)	GSV longit. (MPa)	GSV circ. (MPa)
*E*_ini_	1.63 ± 2.19	2.13 ± 3.10	0.69 ± 0.59	1.15 ± 0.84
*E*_100_	7.50 ± 4.60	9.27 ± 5.35	7.90 ± 3.03	7.27 ± 1.91

### Constitutive modeling

**Fig. 3:**
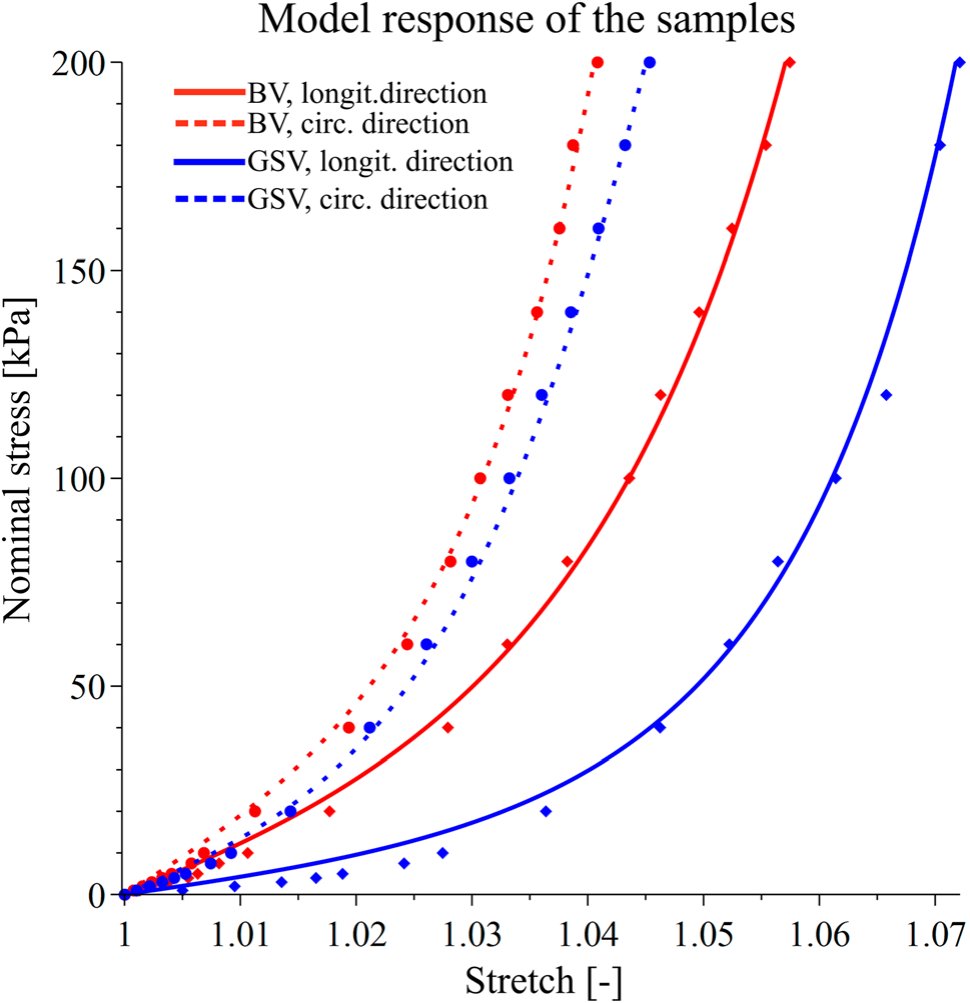
Averaged experimental data (points) and model representation (lines) for donors BV_M77 (red), and SV_M75 (blue) in the longitudinal (solid) and circumferential (dotted) directions

**Fig. 4:**
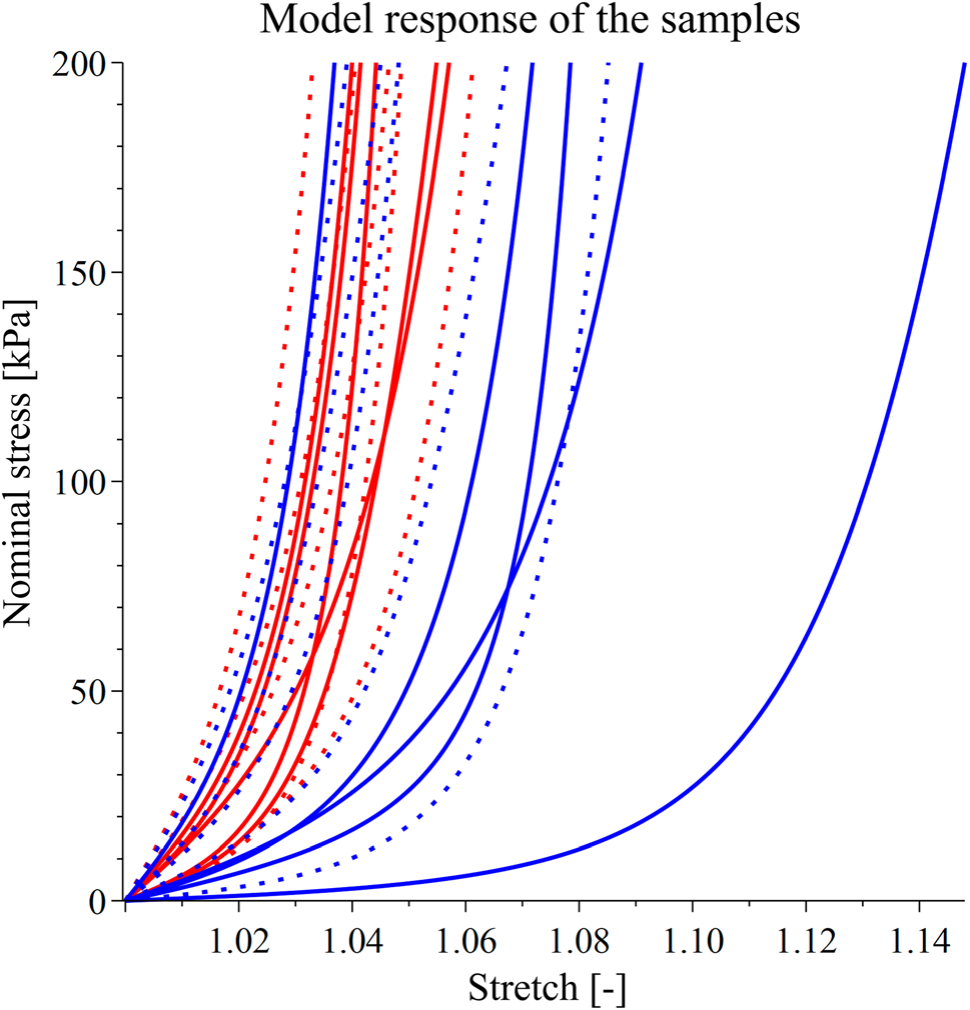
Model mechanical response of the samples. Red—basilic veins, blue—great saphenous veins, solid line—longitudinal direction, dotted line—circumferential direction

The experimental data from strips in the longitudinal and circumferential directions of each donor were averaged and fitted by the hyperelastic constitutive model. The average response of selected samples from each group and its model representation are shown in Fig. 3. A list of constitutive parameter values and the measures of the goodness of fit for all samples are presented in Table 2. Overall, the model was able to fit the data exceptionally well, as suggested by the coefficients of determination *R*^2^, which ranged from 0.97 to 0.99 for the majority of the average curves. The worst fit was obtained for sample SV_75, reaching the value of *R*^2^_circ_ = 0.93. Finally, the model response for all average sample data is shown in Fig. 4. The model response highlights the findings of the higher average stiffness of BVs.

**Table 2:** Constitutive and regression parameters

Sample	No. of specimens	*µ* (kPa)	*k*_1_^1^ (kPa)	*k*_2_^1^ (–)	*k*_1_^2^ (kPa)	*k*_2_^2^ (–)	*k*_1_^3^ (kPa)	*k*_2_^3^ (–)	*β* (–)	*R*^2^_circ_	*R*^2^_longit_
BV_F71	7	1	209.8	221.9	1	88.8	17,418.5	12.9	0.8	0.99	>0.99
BV_F77	8	1	1	1.24	716.2	179.1	937.2	467.9	0.45	0.99	0.99
BV_M77	4	1	284.8	103.6	1	1.1	470	451	1.14	>0.99	0.97
BV_M84	7	1	883.6	141	1	50.2	969.9	500	0.91	>0.99	>0.99
BV_M62	6	1	1	6.2	268.4	253	658.4	309	0.48	0.99	0.98
SV_M71	6	1.47	341.1	160	286.2	96	71.5	87	0.85	>0.99	0.99
SV_M75	5	1.6	267.9	48	15.3	153.9	4773	15.1	0.71	0.93	0.99
SV_M79	8	1	1	97.3	201	82.7	11,926	51.6	0.64	>0.99	0.97
SV_M46	7	1	62.2	88.8	1	17.7	27,481	0.8	0.67	>0.99	>0.99
SV_F76	5	1	1	274.4	232.9	39.2	18,445	46.1	0.59	>0.99	0.98

### Histology

**Fig. 5:**
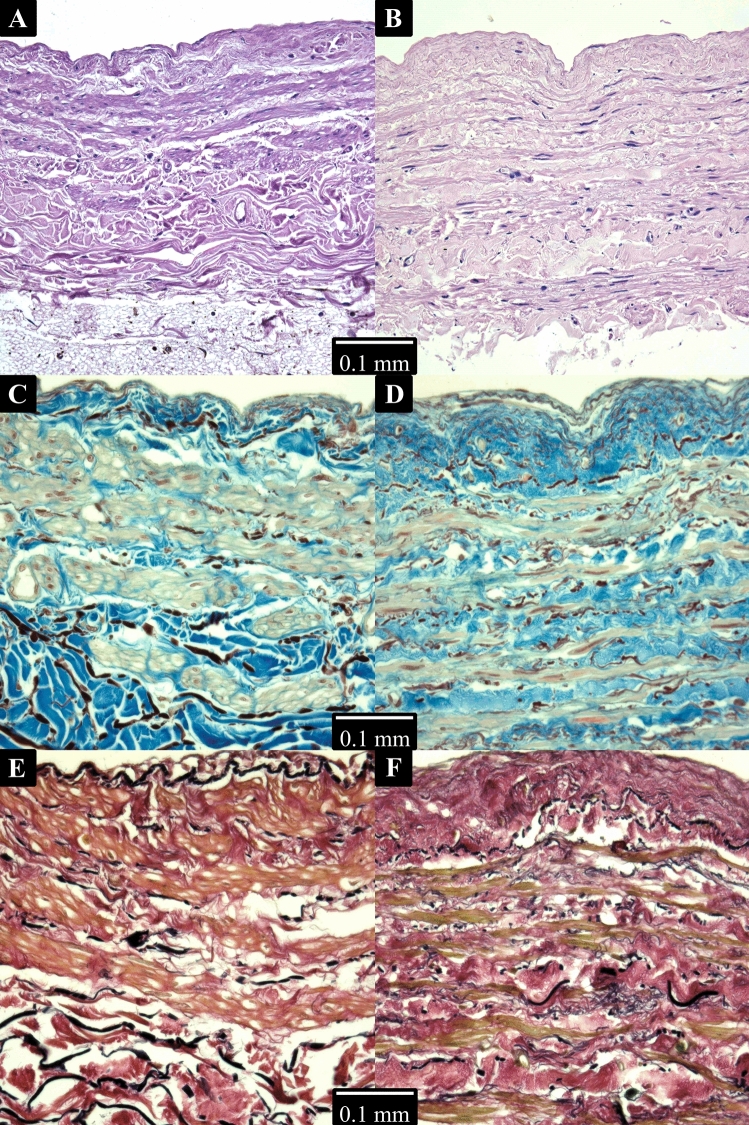
Histology of the samples. **A** BV stained with HE, **B** GSV stained with HE, **C** BV stained with MSB, **D** GSV stained with MSB, **E** BV stained with VG, and **F** GSV stained with VG

Representative micrographs of the vein sections stained for histological analysis are shown in Fig. 5. The basic HE staining showed highly organized tunica media in the GSV. The VSMCs are predominantly oriented circumferentially, which is recognized by the elongated-elliptical shape of their nuclei, stained brown, aligned in that direction. In contrast, the VSMC nuclei of the BV show no clear alignment, taking both elongated and near-circular profiles, suggesting there are fibers oriented in both circumferential and longitudinal directions. The MSB staining provides additional insights, as it can contrastively display collagen, smooth muscle, as well as elastic membranes. The blue-stained collagen in the tunica media of the GSV is more evenly distributed among the beige smooth muscle fibers with darker-stained nuclei, which are oriented circumferentially, showing minimal evidence of other fiber orientations, consistent with the HE staining. Conversely, the visualization of elastic fibers, which appear dark to black in the GSV dyeing, does not reveal differences between the groups. No differences were found in the distribution of acidic mucopolysaccharides using the VG staining.

## Discussion

AVFs are the gold standard for vascular access in patients undergoing hemodialysis, playing a critical role in the management of end-stage renal disease. Despite their widespread use, AVFs face significant challenges regarding maturation and long-term patency, with many requiring further surgical interventions within a few years of employment (Huber et al. 2021; Heindel et al. 2022; Pfister et al. 2023). Since the number of patients undergoing hemodialysis continues to rise globally, improving the long-term functionality and reliability of AVFs remains a key clinical concern.

As a pivotal element of the life-sustaining process of hemodialysis, AVFs have been the focus of extensive research, with numerous studies discussing their functionality, complications, and optimization from both medical and biomechanical points of view. The relationship between the changes in WSS and the loss of patency caused by intimal hyperplasia of the connected vein has been assessed in many studies, reviewed for example in Cunnane et al (2017). Apart from WSS, several other topics have been of major scientific interest, such as general changes in flow parameters (Ventre et al. 2021; Stella et al. 2019; Zhu and Sakai 2021), numerical simulations of the cannulation of the vein (Fulker et al. 2018), and the way the anastomosis angle influences performance of the fistula (Yang et al. 2020). Regarding constitutive description, four approaches of increasing complexity can be used for the tissue: rigid body estimation, linear elasticity, nonlinear elasticity, and the fully viscoelastic approach, potentially including damage modeling. All of these methods have indeed been used in the literature (Huberts et al. 2012; Ng et al. 2023; Zhu and Sakai 2021). However, it has been shown that—while the simpler methods such as rigid body assumption can help to identify places of undesired conditions, for example the high or oscillating WSS—they fail to accurately describe finer details, such as the precise values of the studied parameters.

Decorato et al. (2014) and McGah et al. (2014) assessed the results of the CFD simulations of AVFs with rigid and distensible vessel walls, arriving at the conclusion that the rigid body assumption leads to approximately 10% overestimations of the averaged WSS for blood flow past an arteriovenous fistula. Similarly, Bai and Zhu (2019) demonstrated that apart from the WSS, the disturbance of flow pulsatility is reduced in simulations by incorporating the blood vessel’s elasticity, as studied on an arteriovenous-graft anastomosis. Further, comparing a rigid and a linear elastic model, Marcinnò et al. (2024) report variance in an oscillating shear index pattern. Differences in pressure and velocity values have also been observed in vitro with a rigid model and a compliant 3D-printed model of the fistula (Alam and Newport 2022; Rangel et al. 2023). Among the reasons for adoption of the rigid wall assumption, instead of a more sophisticated approach, is the absence of literature dealing with the constitutive modeling of veins of the upper extremity. And while a model of another blood vessel could be used for a vein of the arm, it should be noted that its mechanical properties might significantly differ between various veins, as documented by Alhosseini Hamedani et al. (2012) who studied human GSVs and umbilical veins. To the best of the authors’ knowledge, there is no study yielding data on BVs suitable for computational biomechanics based on the nonlinear theory.

The mechanical behavior of GSVs observed in this study is consistent with observations reported in the literature (Donovan et al. 1990; Karimi et al. 2015; Veselý et al. 2015; Prim et al. 2016, 2021; Li 2018a, 2018b). As shown in Table 1, the elastic moduli at 100 kPa do not differ significantly from the values reported by Kamenskiy et al. (2011), who found average longitudinal and circumferential tangent elastic moduli of approximately 8 MPa and 6 MPa, respectively, at comparable stress levels. These values fall well within the range observed in the present study.

The results of our study suggest that human BVs are stiffer than GSVs on average, as seen in Figs. 2 and 4, and based on the values in Table 1, although this observation is not universal for all samples. Statistically, the stiffness of the BV does not significantly differ from that of the GSV, given the high variance in the observed behavior. This variance highlights the inter-donor diversity of biomechanical properties, well known in all soft tissues. Nevertheless, while not statistically significant, the difference between the two vein groups seems to be apparent from the model stress–strain curves (Fig. 4). The observed higher stiffness of BVs compared to GSVs contradicts observations made by Eiken and Kölegård (2004). In their study, ultrasonography is used to compare the response of GSVs and cephalic veins as intravascular pressure is increased. This is done by having volunteers put in a pressure chamber, revealing a higher distensibility of the cephalic vein. A possible explanation for this discrepancy is that the authors measured the in vivo distensibility rather than intrinsic mechanical properties, meaning that the surrounding tissue could have influenced the observed behavior. Furthermore, it is possible that the cephalic vein may exhibit properties distinct from the BV despite their physiological likeness. However, it is unlikely that two vessels of similar functions would develop substantial differences in their mechanical behavior. There does not seem to be any literature on the mechanics of basilic veins, specifically disallowing a direct comparison with the results of this study.

A notable difference in the mechanical behavior can be observed in the anisotropy of the vessels. While the circumferential strips of GSVs exhibit a distinctly stiffer initial response than the longitudinal ones, stiffness does not differ in the two directions nearly as profoundly as for BVs, see Table 1. The *p* values computed based on the linear mixed-effect models were *p* = 0.066, and *p* = 0.315, when comparing the initial Young’s moduli in the longitudinal and circumferential directions for GSVs and BVs, respectively. The anisotropy of the GSVs is in line with the observations of other authors on GSVs as well as other veins (Donovan et al. 1990; Alhosseini Hamedani et al. 2012; Karimi et al. 2015), suggesting that the anisotropy of BVs is lower than that of various other vessels.

The anisotropy in the passive mechanical properties is usually contributed to predominant orientation of collagen fibril bundles within the tissue (Holzapfel et al. 2000). However, no significant differences were observed in elastane or collagen orientation in this study. Based on a histological analysis performed on the samples, the lower anisotropy of the BVs could be attributed to less organized muscle fibers, as they were found dispersed in both circumferential and longitudinal directions (Fig. 5). In contrast, the VSMCs of GSVs are predominantly circumferentially oriented. The structure and orientation of the collagen fiber bundles and elastic membranes were similar in both sample groups. This finding suggests that the smooth muscle fibers play a key role in determining the vessel’s stiffness, making it stiffer in the direction of the dominant alignment. The histological features observed in the GSV are in agreement with the literature, as several papers show a similar wall structure (Thiene et al. 1980; Marin et al. 1994; Hashmi et al. 2015). The literature regarding the histology of basilic veins is far scarcer. Rojas et al. (2024) and Kulus et al. (2024) report on the percentage share of muscle, elastic, and collagen fibers in the venous tissue. However, they do not comment on the orientation of the fibers. Their alignment cannot be precisely recognized based on the micrographs provided in the papers. The finding in our study thus seems to be the first reporting the disorganized structure of smooth muscle fibers in BVs.

Apart from an assessment of the mechanical properties of the blood vessels, constitutive modeling of the veins was the main aim of this study. An anisotropic hyperelastic model adopted from Baek et al. (2007) was fitted to the experimental data, following numerous authors who had successfully applied it to model the mechanical properties of other blood vessels (Zeinali-Davarani et al. 2009; Kamenskiy et al. 2017; Jadidi et al. 2020). The 4-fiber family hyperelastic model was capable of fitting the measured data well for both BVs and GSVs, as highlighted by the coefficients of determination in Table 2, and visually shown in Fig. 3. The optimized constitutive parameter value estimates, as given in Table 2, are thus suitable for numerical simulations concerning basilic veins, for example in FSI studies of AVFs. While constitutive modeling of the GSV has been conducted by numerous authors (Zhao et al. 2007; Veselý et al. 2015; Li 2018b), this study is probably the first modeling the BV.

The main limitation of this study stems from the fact that blood vessel samples of the BV and GSV were not obtained from the same five tissue donors. This was due to ethical constraints accompanying the collection of tissues. It is known that significant differences in mechanical properties can be observed in the same blood vessels of various donors (Veselý et al. 2015; Li 2018a). Age is one of the key factors having a major effect on the stiffness of blood vessels (Horný et al. 2014; Kamenskiy et al. 2015; García-Vilana and Sánchez-Molina 2022). Therefore, inter-donor variances in overall health and other conditions that may influence the mechanical properties of their blood vessels are part of the differences observed among the samples. However, nested statistical models revealed that donor-level variance was generally low, with the exception of the longitudinal tangent modulus at 100 kPa, where 31% of the total variance was attributable to differences between donors. For the remaining modulus values, donor-level variance accounted for less than 12%. Thus, the results can provide a valuable insight into the problem, since the age of the donors does not significantly vary between the groups. Furthermore, it would be preferable to conduct the experiments with the specimens submerged in a physiological solution to better resemble the in vivo setting. To approximate the desired conditions as closely as possible, the specimens were kept in saline prior testing. Testing was conducted within seven minutes of their removal from the liquid. Thus, they stayed hydrated throughout the experiment.

It should be noted that cold storage may alter the mechanical properties of vascular tissue. Hemmasizadeh et al. (2012) observed a decrease in the instantaneous Young’s modulus in porcine aortas after 48 h of cold storage. Similarly, Chow and Zhang (2011), using biaxial tensile tests on bovine aortas, reported changes in tangent modulus due to cold storage—specifically, a reduction in initial stiffness and an increase in stiffness at higher stress levels. In contrast, Amin et al. (2011) found no significant differences between fresh and 48-h cold-stored mouse carotid arteries using inflation tests. These ambiguous results suggest that the effects of short-term cold storage on vascular mechanics remain unclear. Therefore, while the 48-h delay between sample acquisition and mechanical testing in our study may introduce some uncertainty, it can be expected that our results will not be too far from those we would have obtained if we had been able to completely eliminate the delay between tissue collection and the experiment.

Biaxial testing would be preferable regarding constitutive modeling of the tissue, as 2D stress state would probably simulate in vivo loading conditions more closely. While biaxial planar experiments were considered for this study, their implementation proved to be technically infeasible given the small dimensions of the vessels. However, the 4-fiber family model was reported to have good 2D stress–strain response predicting capabilities, implementing parameter values obtained in uniaxial experiments (Schroeder et al. 2018).

## Conclusion

Uniaxial tensile experiments were carried out with samples of human GSVs and BVs. Data from the experiments was subjected to regression analysis aimed at constitutive modeling of the samples. An anisotropic hyperelastic model was successfully employed to fit the measured mechanical response. The obtained estimates of constitutive parameters can be used in numerical simulations of mechanical interactions within BVs, for example in studies dealing with the mechanobiology of the AVFs used as vascular access for hemodialysis.

## Data Availability

Research data are available via 10.5281/zenodo.15473182.
